# Practical Application of Columbia Classification for Focal Segmental Glomerulosclerosis

**DOI:** 10.1155/2016/9375753

**Published:** 2016-05-09

**Authors:** Man-Hoon Han, Yong-Jin Kim

**Affiliations:** ^1^Department of Pathology, Kyungpook National University Hospital, 130 Dongdeok-ro, Jung-gu, Daegu 41944, Republic of Korea; ^2^Department of Pathology, Yeungnam University College of Medicine, 170 Hyunchung-ro, Nam-gu, Daegu 42415, Republic of Korea

## Abstract

Focal segmental glomerulosclerosis (FSGS) is a heterogeneous clinicopathological entity. Two frameworks for the classification of FSGS have been described: etiologic and morphologic. The etiologic classification is distinguished among genetic, adaptive, virus-associated, drug-induced, and idiopathic types. Morphologic classification is commonly referred to as the Columbia classification published in 2004, which distinguishes five variants: collapsing, tip, cellular, perihilar, and not otherwise specified (NOS). This classification is based on light microscopic patterns with rigorously defined specific criteria, which can be applied to primary and secondary forms of FSGS, and has been widely used over the past 10 years both as a diagnostic and as a prognostic clinical tool. This paper defines common histopathological features of FSGS, distinguished characters among five variants, and points out the confusion about terminology of variants, because most were proposed in the past with different definitions. Despite good interobserver reproducibility of this classification system, difficulty in its application may arise in the interpretation of lesions with mixed features of more than one variant in the same tissue specimen and with late lesions, because other variants may evolve into the NOS variant over time.

## 1. Introduction

Focal segmental glomerulosclerosis (FSGS) is the name of the primary glomerular disease as well as the terminology describing the secondary scar phenomena by injury of other glomerular diseases. Since the first descriptions by Fahr and Rich, several different histologic variants of FSGS have been described [1]. Histologically, it is characterized by sclerosis, hyalinosis, foam cell infiltration, vacuolization of podocytes, and podocyte proliferation. Mixed use of the term FSGS and heterogeneous morphologic features cause confusion both in making a diagnosis and in correlating with underlying pathogenesis. We attempted to clarify the morphologic terminologies for featuring FSGS and described practical application of the Columbia classification [2] and discussed confusion points of previous subtypes: tip, cellular, and collapsing variants.

## 2. Common Histologic Features

### 2.1. Focal and Segmental Lesions (Figure 1)

“Focal” is defined as a focal lesion that affects some glomeruli. Thus, occasionally, only one sclerotic glomerulus can be found despite a diligent search in a renal biopsy specimen [3]. “Segmental” is defined as a lesion partially involving a single glomerulus. The unaffected glomeruli show normal finding. In case of absence of sclerotic glomeruli in a renal biopsy, differentiation of FSGS from “minimal change disease” (MCD) is difficult.

### 2.2. Sclerosis and Hyalinosis (Figure 2)

Sclerosis, the representative and typical change of FSGS, is a vascular change showing stiffness and obstruction similar to arteriosclerosis. As sclerosis progresses, proteinaceous material resulting from plasmatic insudation may be found in sclerotic glomerulus. The proteinaceous material shows a glassy pink appearance in H&E stain and is thus called “hyalinosis.” This lesion turns pale pink in periodic acid-Schiff (PAS) stain and dark red in trichrome stain. Hyalinosis was considered a characteristic lesion of FSGS in the past; thus, the term “focal segmental hyalinosis” was used. It is still used together with FSGS, occasionally [1]. Because they have morphologic similarity, differentiation of a scar from sclerosis sometimes can be difficult. Therefore, it is necessary to examine the nonsclerotic glomeruli and check the clinical features for differential diagnosis from a scar.

### 2.3. Vacuolization of Podocytes

Vacuoles may be observed in cytoplasm of podocytes as a result of damage. This lesion is considered evidence that FSGS is attributable to damage of podocytes. Vacuolization is more clearly observed using the electron microscope.

### 2.4. Halo Formation (Figure 3)

Podocytes in involved glomeruli may be detached from the glomerular capillary basement membrane. The space between the podocyte and the glomerular capillary basement membrane is filled with new collagen fiber. In trichrome stain, the collagen fiber shows a paler blue than other capillaries. This seems like the appearance of the “halo” of the moon and is observed better using the electron microscope.

### 2.5. Distribution and Location of Sclerosis

**Figure 4:**
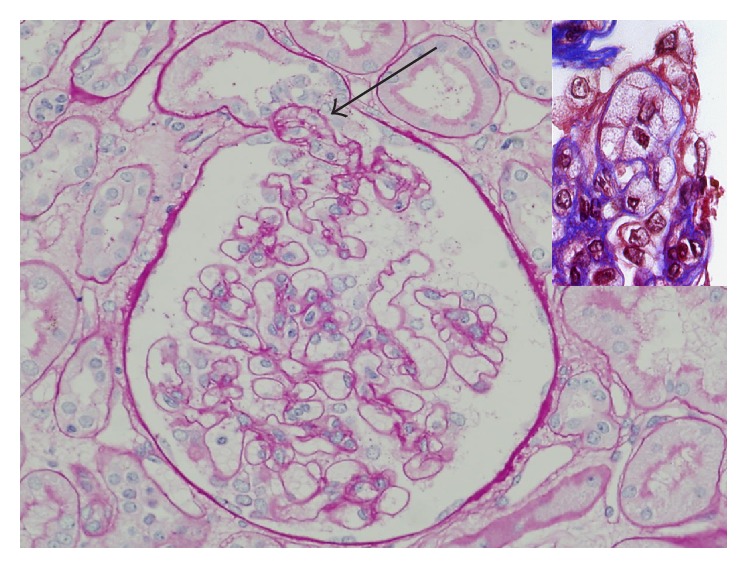
Tip variant of FSGS. Foam cell accumulated segment prolapsed into the tubular pole, the origin of the proximal tubule. The remainder of the glomerular tuft appears normal. PAS stain, ×200. (Courtesy of Professor Mi Sun Choi, DongSan Hospital of Keymyung Medical College, Daegu, Korea.) Inset shows endocapillary foam cells in trichrome stain. ×400.

In early FSGS, only a few glomeruli are involved, and these show small sclerotic lesions. Sclerosis initially occurs in the juxtamedullary area [4]. This is believed to be due to the fact that the juxtamedullary area shows high blood pressure and high blood flow [5]. Glomeruli in upper cortex are involved last. If the corticomedullary junction is not included in a biopsy specimen, the diagnosis of FSGS would likely be missed. In one glomerulus, sclerosis occurs in a peripheral area rather than center and adhesion between Bowman's capsule and sclerotic lesion is often exhibited. Correlation between distribution of sclerosis and prognosis has been studied for a long time. The tip lesion (Figure 4), which occurs in an adjacent area to the origin of the proximal tubule, is noted for showing the most favorable prognosis [6, 7]. However, as sclerosis progresses, it is difficult to determine the original location of sclerosis.

### 2.6. Global Sclerosis

As segmental sclerosis is progressed, entire glomerulus becomes involved. Terminal FSGS shows global sclerosis in most glomeruli. However, because global sclerosis may normally occur with age, it should not be concluded that FSGS would be somewhere [8, 9].

### 2.7. Glomerulus without Sclerosis (Unaffected Glomerulus)

In cases of unaffected glomerulus, due to normal findings (Figure 4), it cannot be differentiated from MCD. However, in morphometric study, it was proved that glomerulus showing no sclerosis in FSGS was increased slightly compared to the normal size for the age [10]. Therefore, when the size of glomerulus is larger than normal, FSGS should be considered, even if there is no renal glomerulosclerosis in the biopsy specimen.

### 2.8. Hypercellularity

**Figure 5:**
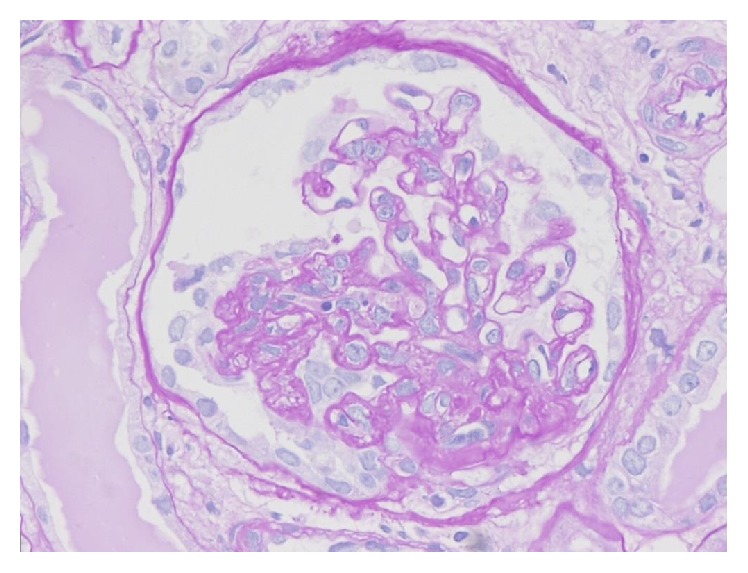
Collapsing variant of FSGS. Segmental collapse of glomerular capillaries is accompanied by proliferation of overlying podocytes. PAS stain, ×200.

**Figure 6:**
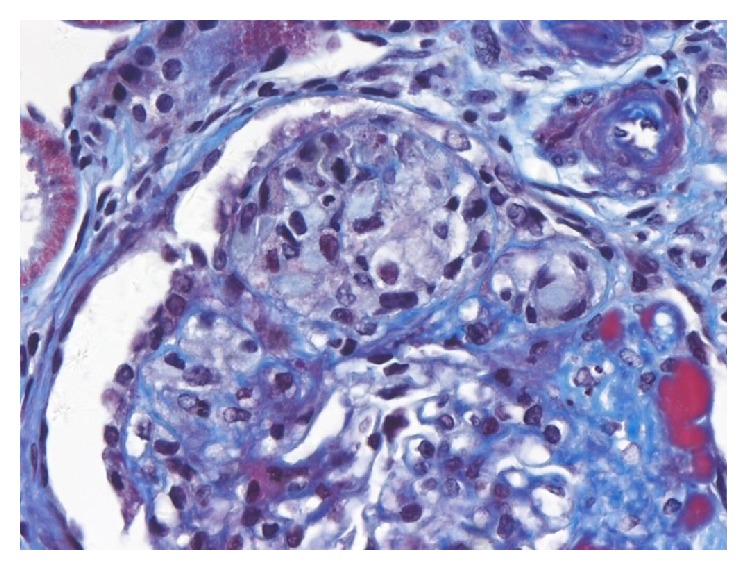
Cellular variant of FSGS. Segment is expanded by endocapillary foam cells. Overlying epithelial cells are also prominent, but capillary collapse is not observed. Trichrome stain, ×200.

FSGS is essentially a nonimmunologic disease; therefore, cell proliferation is not the fundamental lesion in FSGS. Mesangial cell proliferation, believed to be a feature of FSGS in the past, can be confused with other diseases including like IgA nephropathy with FSGS pattern. Proliferation of podocytes may be found in FSGS. In 1985, Schwartz and Lewis classified these lesions as a feature of proliferative FSGS [11]. However, it has since been excluded from the cellular type and recognized as characteristic changes in the collapsing type (Figure 5) [2]. In cases involving severe proliferation of podocytes, it is often confused with the crescent. Endocapillary proliferation can also occur in FSGS. Excessive infiltration of inflammatory cells and many foam cells are recognized as features of cellular type of FSGS (Figure 6). Foam cells are considered as vascular endothelial cells or monocytes including mainly fat of the plasma component.

### 2.9. Tubules and Interstitium (Figure 1)

Focal tubular atrophy, interstitial fibrosis, and lymphocytic infiltration are features of FSGS. The severity of these lesions is associated with the severity and the number of involved glomeruli, but it is not necessarily proportional to the severity. Most MCD do not show these changes in tubules and interstitium. Therefore, these are important findings that are more consistent with FSGS rather than MCD, particularly in cases of suspicious FSGS clinically but no definite sclerotic lesion was found in a biopsy specimen [8, 9].

### 2.10. Vessel (Figure 1)

Findings such as arterial and arteriolar nephrosclerosis, accompanied by high blood pressure, appear in the blood vessels of FSGS (i.e, the thickening of arterial wall, intimal fibrosis, and sometimes subendothelial hyaline deposition). As these findings become severe, glomerulosclerosis and the damage of interstitium and tubules become more severe, resulting in greater reduction of renal function.

### 2.11. Immunofluorescence Findings

Because FSGS is essentially a nonimmunologic disease, immunologic deposition is not present in FSGS in principle. However, deposition of C3 and IgM is sometimes found in the site of sclerosis, particularly in the site where hyaline material is deposited [6]. That is not attributable to the immunologic reaction and is a nonimmunologic and nonspecific finding as the result of combination with the absorbed or retained plasma protein. Thus, IgM, the most common among immunoglobulin, and C3 are demonstrated in the lesions [3]. In unaffected glomeruli, there is no deposition and, even if there is, it is nearly negligible.

### 2.12. Electron Microscopic Findings

The sclerotic segment shows wrinkled basement membrane and foamy macrophages containing bubble-shaped protein and lipid droplets. Increased electron density, a result of fusion of wrinkled basement membrane, may sometimes be mistaken for the immune-type deposit. However, it shows no clear shape of electron dense deposits in certain types of immune complex glomerulonephritis and is not present in nonsclerotic area [3]. Effacement of foot processes, mainly observed in MCD, is seen in unaffected areas. Although the severity of effacement of foot processes is variable according to the amount of urinary protein excretion, it is generally less in FSGS than in MCD. This lesion is not discriminated between FSGS and MCD, but in FSGS, unlike MCD, vacuolization and proteinaceous material are frequently found in cytoplasm of podocytes.

## 3. Application of Columbia Classification (Table 1)

Two frameworks for the classification of FSGS can be described: etiologic and morphologic. The first, etiologic classification, is distinguished among genetic, adaptive (hyperfiltration), virus-associated, drug-induced, and primary or idiopathic types [12]. The second, morphologic classification, is commonly referred to as the Columbia classification published in 2004, which describes five distinct FSGS variants: collapsing, tip, cellular, perihilar, and not otherwise specified (NOS), which is based on light microscopic patterns [13] and has been widely used over the past 10 years both as a diagnostic and as a prognostic clinical tool. This classification system can be applied to both primary and secondary forms of FSGS but should not be confused with pathogenic mechanisms in the development of that defined lesion. On the other hand, in the Columbia classification each variant of FSGS is rigorously defined by specific criteria, tremendously reducing the confusion about the terminology that characterized the last two decades of the 20th century.

### 3.1. Collapsing Variant (Figure 5)

This type is characterized by the presence of at least one glomerulus with collapse and overlying podocyte hypertrophy and hyperplasia, regardless of the presence of other lesions resembling the other four variants of FSGS [13]. Thus, the finding of a single collapsing lesion trumps all other variants. Podocyte proliferation within Bowman's space sometimes has a “pseudocrescent-like” feature. Collapsing lesions are more commonly global than segmental and are often accompanied by severe tubulointerstitial injury with microcysts and hypertrophic tubular epithelial cells swollen with hyaline protein reabsorption droplets. Foot process effacement is usually diffuse. Most cases are either idiopathic in origin or HIV-associated and are more commonly found in black patients [14, 15].

### 3.2. Tip Variant (Figure 4)

Diagnosis of tip variant, after excluding collapsing and perihilar lesions, requires at least one segmental lesion involving the “tip” domain, the outer 25% portion of the glomerular tuft next to the origin of the proximal tubule with either extracellular matrix adhesion or confluence of podocytes with parietal or tubular epithelial cells at the tubular lumen or neck [13]. Tip lesions are typically cellular (81%) and contain prominent endocapillary foam cells, but they may be sclerosing and contain hyaline [16]. The tip variant typically shows only mild chronic tubulointerstitial injury and arteriosclerosis [2]. Foot process effacement is typically severe and diffuse. Most cases of tip lesion FSGS are idiopathic in etiology and predominate in white adults [17]. Most pure tip lesions have a very good prognosis and response to steroid therapy [2, 18].

In the early description of the glomerular “tip lesion” by Howie and Brewer [6], it was not restricted to FSGS but rather a novel report of a curious glomerular abnormality seen independently in patients with proteinuria and was also found in other heterogeneous renal abnormalities with associated proteinuria, including membranous glomerulopathy and diabetic glomerulosclerosis. However in Columbia classification tip variants contained nontip segmental lesions (75%) involving the periphery of the tuft [10]. Thus, the presence of a nontip lesion of this variant differs from the original description. And, because it may occur in association with other glomerular diseases, the question among renal pathologists is whether a tip lesion simply represents a protrusion of the tip of the glomerulus into the tubular pole and a nonspecific glomerular abnormality in response to proteinuria or a variant of MCD [18, 19].

### 3.3. Cellular Variant (Figure 6)

This is defined by identification of at least one glomerulus with endocapillary hypercellularity (including foam cells, macrophages and other leukocytes, and endothelial cells, occasionally associated with hyalinosis, karyorrhexis, and fibrin) involving more than 25% of the glomerular tuft, leading to occlusion of the capillary lumen [13]. Because foam cells may be seen in other FSGS subtypes, the diagnosis requires exclusion of tip lesion and collapsing variants [6]. Cellular lesions are typically found in the peripheral tuft [2]. This variant may lack any evidence of segmental scars, mimicking a focal proliferative glomerulonephritis. Foot process effacement is usually severe [2]. This is the least common variant but has poor prognosis [20].

In contrast with the original description of a cellular lesion [21], the Columbia classification restricts the hypercellularity to the endocapillary compartment of the glomerulus and does not (as originally reported) occur with collapse of the glomerular basement membranes. Therefore, podocyte abnormalities are not a defining feature for the cellular variant. Stokes and D'Agati [2] pointed out a problem in recognition of cellular lesions because endocapillary foam cells are not a specific feature, but they may occur to some degree in other variants. What distinguishes the cellular variant is the expansile, purely cellular nature of the endocapillary lesions which typically lack appreciable extracellular matrix.

### 3.4. Perihilar Variant (Figure 2)

The variant is defined by the presence of at least one glomerulus with perihilar hyalinosis with or without sclerosis and sclerotic lesions at the glomerular vascular pole (perihilar) in more than 50% of affected sclerotic glomeruli [13]. Tip lesion, collapsing, and cellular variants must be excluded [13]. This form has been described in both primary FSGS and secondary adaptive forms stemming from nephron loss or glomerular hypertension (i.e., due to obesity, reflux nephropathy, hypertension, and sickle cell disease), usually accompanied by glomerular hypertrophy. In the adaptive conditions, reflex dilatation of the afferent arteriole leading to glomerular hypertension may cause particular stress on the perihilar segment [20]. Foot process effacement is usually focal and relatively mild.

### 3.5. FSGS NOS Variant

Finally, this applies to a renal biopsy that does not meet the criteria for any other variant with findings of focal and segmental consolidation of the glomerular tuft by increased extracellular matrix, leading to obliteration of glomerular capillary lumen [13]. This is the most common subtype, and, interestingly, it has been observed from repeat biopsies that other variants may evolve into FSGS NOS over time [17, 22].

## 4. Limitation of Classification

In general, the detection of a FSGS depends on the percentage of glomeruli affected, the size of the segmental lesions, and the number of serial sections studied. Lesions may be lost during histologic preparation as well as due to sampling error, leading to underdiagnosis. Thus, biopsy size is also an important component of assessment of focal lesions. It is estimated that a minimum of 25 glomeruli is necessary to detect a low prevalent lesion [17]. Multiple sectioning may be necessary especially to define tip lesions or cellular variant. Of even further importance, the biopsy must include the juxtamedullary region. Because some FSGS variants, including perihilar type, initially started from juxtamedullary glomeruli, superficial cortical biopsy samples may not contain lesions. Despite good interobserver reproducibility of this classification system [23], difficulties in application of this classification may arise in the interpretation of lesions with mixed features of more than one Columbia type of FSGS in the same tissue specimen. In addition, because other variants may evolve into the NOS variant over time, such variants may be less frequent in biopsies obtained late in the disease course. Thus, the spectrum of FSGS lesions likely includes dynamics related to time of biopsy, as well as divergence of initial pathogenic insults. The histologic phenotype, thus, gives clues to both stage and type of initial injury [24].

Barisoni et al. [25] suggested that collapsing glomerulopathy and glomerular tip lesion should be classified separately from FSGS because of the lack of sclerosis. However, it is reasonable to include them within the spectrum of FSGS, because both of these entities demonstrate segmental glomerular lesions and may be accompanied by “classic” FSGS lesions [17]. And the findings of different clinical characteristics in both variants suggest that these morphologic variants reflect distinct biological pathways regardless of etiology [2].

This classification is only based on glomerular changes and does not mandate tubulointerstitial injury, global glomerulosclerosis, interstitial inflammatory changes, or degree of effacement of foot processes in electron microscopic changes. All those have been considered as prognostic indices. Further study of the biopsy, including immunohistochemistry and electron microscopy, may help in further understanding the spectrum of segmental lesions. And recent tremendous advances in discovery of genetic and molecular mechanisms of renal diseases are, indeed, the case of FSGS [26–29]. This group of authors recently suggested a new approach to classification of diseases with primary FSGS that integrates conventional histologic features with etiology.

## 5. Conclusion

Ideally, classification of a disease should be based on pathogenesis and/or etiology, but the Columbia classification is just based on light microscopic morphologic changes. However, when we think about the fact that at the time of biopsy, usually, causes of FSGS in patients were unknown, this morphologic classification has been a useful working proposal to identify subgroups. Using this morphologic classification, significant differences in baseline clinical characteristics and outcomes among the variants of had been demonstrated in several studies, especially tip and collapsing variants [2, 20, 24].

For further identification of clinical significance, morphologic studies including tubulointerstitial changes and findings of immunohistochemistry and electron microscopy would be necessary. And a goal of future investigations should be to correlate morphologic variants with etiology especially genetic factors.

## Figures and Tables

**Figure 1:**
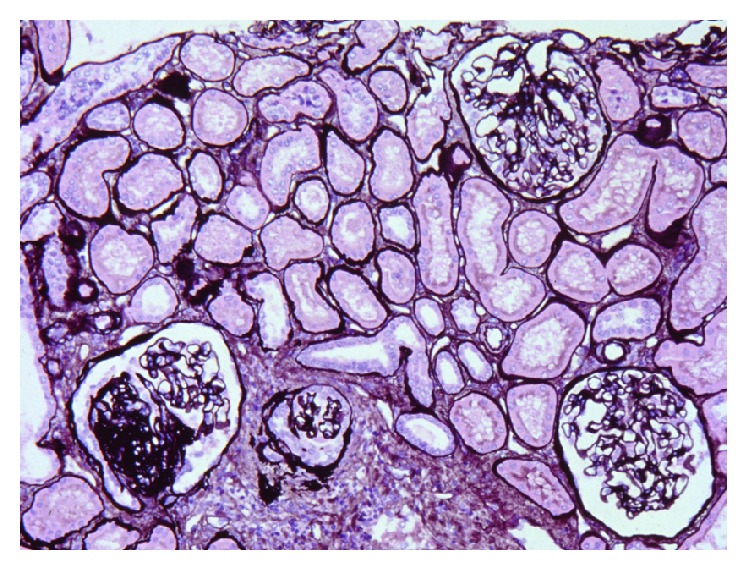
Segmental sclerotic glomerulus is at left lower and right two glomeruli look normal. Interstitial fibrosis and tubular atrophy are observed focally. Artery shows intimal fibrosis. Jones methenamine silver stain (PAM), ×200. (Permitted by the Journal of Korean Society of Pediatric Nephrology [3].)

**Figure 2:**
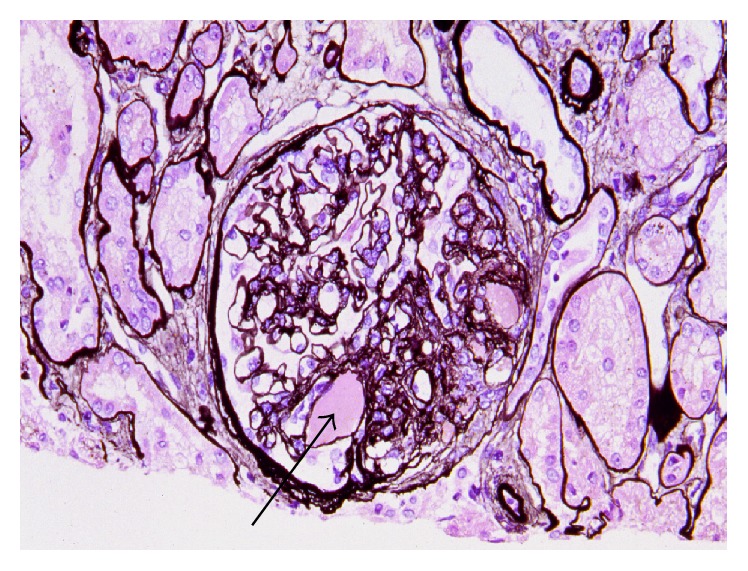
Perihilar variant of FSGS. Sclerosis is observed at the glomerular vascular pole. Hyaline (arrow) is the amorphous material in the middle of sclerosis, PAM, ×200. (Permitted by the Journal of Korean Society of Pediatric Nephrology [3].)

**Figure 3:**
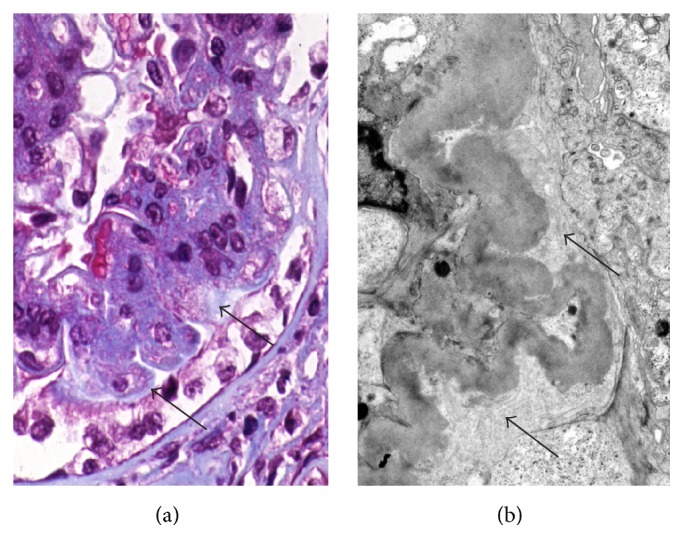
Halo formation. In trichrome stain (a), the pale zone (arrow) is between sclerosis and overlying podocytes. Trichrome stain, ×400. Some areas on the electron micrograph (b) are filled with newly formed thin collagen bundles (arrows). ×5000. (Permitted by the Journal of Korean Society of Pediatric Nephrology [3].)

**Table 1:** Columbia classification of FSGS variants.

Variant	Inclusion criteria	Exclusion criteria
FSGS (NOS)	At least 1 glomerulus with segmental increase in matrix obliterating the capillary lumina. There may be segmental glomerulus capillary wall collapse without overlying podocyte hyperplasia.	Exclude perihilar, cellular, tip, and collapsing variants.

Perihilar variant	At least 1 glomerulus with perihilar hyalinosis, with or without sclerosis. >50% of glomeruli with segmental lesions must have perihilar sclerosis and/or hyalinosis.	Exclude cellular, tip, and collapsing variants.

Cellular variant	At least 1 glomerulus with segmental endocapillary hypercellularity occluding lumina, with or without foam cells and karyorrhexis.	Exclude tip and collapsing variants.

Tip variant	At least 1 segmental lesion involving the tip domain (outer 25% of tuft next to origin of proximal tubule). The tubular pole must be identified in the defining lesion. The lesion must have either an adhesion or confluence of podocytes with parietal or tubular cells at the tubular lumen or neck. The tip lesion may be cellular or sclerosing.	Exclude collapsing variant. Exclude any perihilar sclerosis.

Collapsing variant	At least 1 glomerulus with segmental or global collapse and overlying podocyte hypertrophy and hyperplasia.	None.

FSGS, focal segmental glomerulosclerosis; NOS, not otherwise specified.

Reprinted from [13].
